# A biocompatible NIR-II light-responsive nanoknife for permanent male sterilization†

**DOI:** 10.1039/d3na00189j

**Published:** 2023-08-16

**Authors:** Haoyu Wang, Xiaomeng Yue, Huanhuan Wu, Yeda Wan, Yujie Tong, Yang Zhao, Yijun Li, Jinbin Pan

**Affiliations:** a Department of Radiology, Tianjin Key Laboratory of Functional Imaging, Tianjin Medical University General Hospital Tianjin 300052 China panjinbin@tmu.edu.cn; b Department of Radiology, Affiliated Hospital of Inner Mongolia Medical University Hohhot 010050 China; c Ultrasonic Diagnosis and Treatment Department, National Clinical Research Center of Cancer, Tianjin Cancer Hospital Ariport Hospital Tianjin 300052 China; d Department of Radiology, The Second Hospital of Tianjin Medical University Tianjin 300211 China; e Inner Mongolia Medical University Hohhot 010050 China

## Abstract

Nanomaterial-mediated photothermal therapy (PTT) is a promising strategy for permanent male sterilization owing to its easy operation, rapid heating, minimal invasiveness, and high spatiotemporal controllability. However, the currently available PTT for male sterilization utilizes irradiation sources in the first near-infrared window (NIR-I), which may suffer from incomplete sterilization due to the insufficient penetration depth of NIR-I light. Herein, we developed a facile one-pot hydrothermal synthetic method of cysteine-coated copper sulfide (Cys-CuS) nanosheets for the second NIR window (NIR-II) PTT-mediated permanent male sterilization. In this method, Cys acted not only as a template but also as a sulfur resource in the formation of Cys-CuS nanosheets. The obtained Cys-CuS nanosheets possessed good photothermal properties and satisfied deep-tissue light response capacity under 1064 nm laser exposure. Given this, the permanent male sterilization *in vivo* was readily achieved by Cys-CuS nanosheets in a rapid manner (only 40 s). To the best of our knowledge, it is the first time that nanomaterial-mediated NIR-II PTT is applied for permanent male sterilization. We believe that the facilely prepared biocompatible Cys-CuS nanosheets can serve as a promising NIR-II light-responsive nanoknife to control the overpopulation of domestic pets and stray animals.

## Abstract 2

A NIR-II light-responsive “nanoknife” (Cys-CuS nanosheets) to achieve high-efficiency permanent male sterilization.

## Graphical abstract

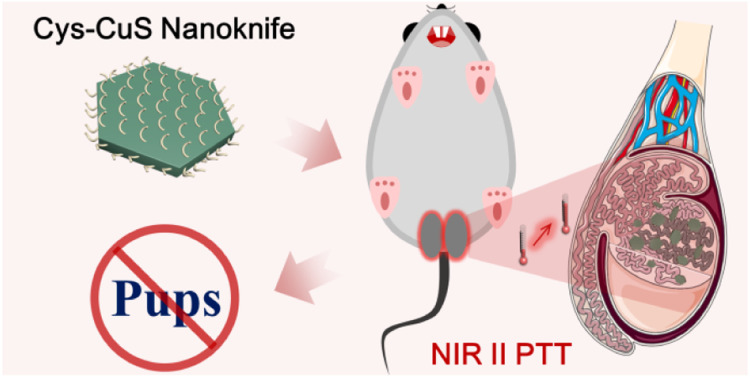

## Introduction

Permanent male sterilization has emerged as a critical tool to control the overpopulation of domestic pets and stray animals.^1^ Traditional methods such as surgical operation, chemical injection, as well as anti-fertility vaccines, have shown promising outcomes for male sterilization.^2,3^ However, these methods still suffer from some drawbacks in practical applications.^1^ For example, the surgery is limited by invasive procedures and risks of postoperative infection.^4,5^ Whereas, the injection of chemical sterilants or vaccines may induce long-term immunosuppression and adverse reactions.^6^ Therefore, a simple, controllable, more effective, and less invasive permanent male sterilization method is highly desirable to overcome the above-mentioned limitations.

In fact, the testicle is a vulnerable organ sensitive to high temperature, and hyperthermia can easily destroy testicular function, injure spermatogenesis, and induce male infertility.^7,8^ Inspired by this theory, an alternative strategy for male sterilization *via* nanoparticles-mediated hyperthermia under light irradiation or alternating magnetic field is gaining attention.^1,9,10^ In particular, photothermal therapy (PTT), based on local thermal ablation induced by photoabsorbers under irradiation of near-infrared (NIR) light, is a promising method for male sterilization owing to its easy operation, rapid heating, minimal invasiveness, and high spatiotemporal controllability.^11–13^ Currently, several photothermal absorbers (PTAs) such as gold nanorods,^11^ tungsten oxide,^14^ and plasmonic copper sulfide^9^ have been successfully applied in animal experiments for PTT male sterilization. However, these studies utilized irradiation sources just in the first NIR window (NIR-I, 650–950 nm), which may suffer from incomplete sterilization due to the insufficient penetration depth of NIR-I light. By contrast, PTT induced by a second NIR window (NIR-II, 1000–1700 nm) is considered much more attractive in thermal ablation with great superiorities in deeper penetration, lower tissue self-heating, and larger maximum permissible exposure in living systems.^15–19^ Moreover, the wavelength range of 1000–1100 nm possesses maximal penetration depth in biological tissue, which is more likely to satisfy the deep-tissue PTT.^20^ Hence, it is highly attractive to apply NIR-II PPT for permanent male sterilization to avoid the omission of deep testicular tissue and totally destroy the fertility ability.

Among various PTAs, copper sulfide nanoparticles (CuS NPs) have garnered our attention due to their powerful localized surface plasmon resonance covering both NIR-I/II windows and their impressive photothermal conversion ability.^21–26^ Furthermore, Cu ions degraded from CuS NPs are essential microelements in the human body, showing good biocompatibility. However, to prepare suitable CuS NPs for bioapplications, further modification is indispensable after the formation of nanocrystals in classic synthetic routes,^27^ which is often complexed with extra procedures and not convenient for large-scale industrial preparation. Recently, biomolecule-assisted biomineralization synthesis has been considered a green and efficient method for preparing biocompatible CuS NPs. Our group has previously reported the synthesis of protein–CuS NPs using albumin and Na_2_S as a template and sulfur source, respectively.^28^ In addition, Liu *et al.* designed a general method to obtain metal sulfides for cancer PTT, in which the protein worked not only as a template but also as a sulfur donor to achieve metal sulfide nucleation.^29^ In this strategy, the S-rich cysteine (Cys) residuals in protein are the key points, which could convert into active sulfur anions and react with metal ions. However, the large content of protein present in the formed CuS NPs would limit its photothermal conversion performance. Compared with Cys-contained protein, Cys itself has great potential in serving as a more promising precursor to fabricate metal sulfide nanocrystals *via* a simple and green method without extra sulfur resources.^30,31^ Therefore, it is appealing to use Cys as both template and sulfur resource to prepare CuS NPs with good NIR-II photothermal performance and desirable biocompatibility, and explore its potential for NIR-II light-responsive permanent male sterilization.

**Scheme 1:**
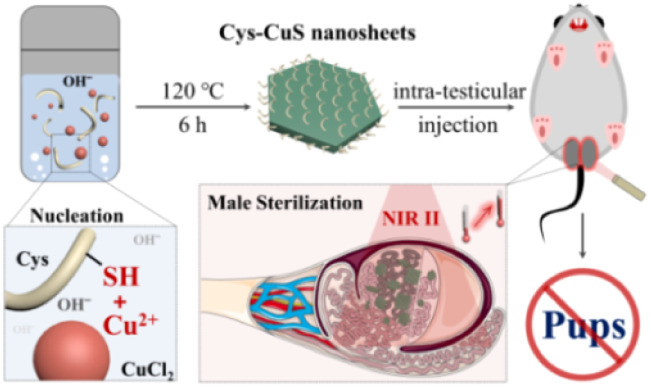
Schematic illustration of the preparation of Cys-CuS nanosheets as a biocompatible NIR-II light-responsive photoabsorber for permanent sterilization.

Herein, we reported a facile one-pot hydrothermal synthetic method of Cys-coated CuS nanosheets for NIR-II PTT-mediated permanent male sterilization for the first time. In this method, S-rich Cys could seize Cu^2+^ to form Cys-Cu^2+^ complexes and participate in the nucleation of CuS under alkali and heated conditions. The endogenous Cys-mediated aqueous-phase synthesis also minimized the potential biotoxicity of CuS nanosheets. The obtained polygon Cys-CuS nanosheets exhibited strong and broad absorption in both NIR-I and NIR-II windows and satisfactory photothermal conversion efficiency and extinction coefficient under irradiation of a 1064 nm laser. Benefiting from the admirable NIR-II photothermal performance of Cys-CuS nanosheets, rapid permanent male sterilization *in vivo* was easily realized (Scheme 1). We believe that this work not only identifies a facile synthetic method of CuS nanomaterials for NIR-II PTT but also provides a promising biocompatible tool to control the overpopulation of domestic pets and stray animals.

## Experimental

### Chemicals

All chemicals and reagents used in this work were at least of analytical grade and used without further purification. Copper chloride (CuCl_2_), l-cysteine, sodium hydroxide (NaOH), and dinitrosalicylic acid (DNS) were provided by Aladdin Chemistry Corporation (Shanghai, China). Propidium iodide (PI) and calcein acetoxymethyl ester (Calcein-AM) were obtained from Dojindo (Shanghai, China). DMSO was purchased from Concord Technology (Tianjin, China). Rat testosterone (T) ELISA kit was purchased from Biotech Bioengineering (Shanghai, China) Co., Ltd.

### Synthesis of Cys-CuS nanosheets

l-Cysteine (60.5 mg) was previously dissolved in deionized water (16 mL). Then, CuCl_2_ solution (1 mL, 50 mM) was mixed with the above solution under magnetic stirring and kept stirring at room temperature for half an hour. Subsequently, NaOH solution (1 M, 250 μL) was added dropwise, and the white mixed solution gradually turned colorless. After stirring for another 30 min, the mixture was transferred to a Teflon-lined stainless-steel autoclave (25 mL), followed by heating at 120 °C for 6 h in the air oven. After naturally cooling to room temperature, the mixture was arranged with purification by dialysis against deionized water for 24 h (the molecular weight cut-off of the membrane was 8000–14 000). Finally, Cys-CuS nanosheets were obtained using a freeze-drying method and kept at 4 °C for further study.

### Characterizations

The X-ray diffraction (XRD) pattern was obtained on a D/MAX-2500PC X-ray diffractometer (Rigaku, Japan). To identify the chemical state of Cu and S in the Cys-CuS nanosheets, X-ray photoelectron spectroscopy (XPS) measurements were performed on an Axis Ultra DLD spectrometer (Kratos Analytical, Manchester, UK). HRTEM was performed on a Philips Tecnai G2 F20 (Phillips, The Netherlands) instrument. UV-vis-NIR absorption spectra were recorded on a UV-3600 spectrophotometer (Shimadzu, Japan). Zeta potential and dynamic light scattering (DLS) were obtained on a Malvern Zetasizer (Nano series ZS, UK).

### Stability assessment

For the stability assessment, the obtained Cys-CuS nanosheets were stored under 4 °C as powder. The UV-vis-NIR absorption spectra of Cys-CuS nanosheets (50 mg L^−1^) were obtained at different time points (1, 3, 5, 7, and 18 days).

### Photothermal performance

To investigate the photothermal performance of CuS nanosheets, different concentrations of Cys-CuS nanosheets solutions (1 mL; 50, 100, and 200 mg L^−1^) were added into a quartz cell and subsequently irradiated using a 1064 nm laser (2 W cm^−2^, 5 min). At the same time, the infrared thermal images were recorded using a FLIR E75 infrared camera. To assess the photothermal stability, Cys-CuS nanosheet solution (100 mg L^−1^) was irradiated with a 1064 nm laser (2 W cm^−2^) for 5 min and naturally cooled to room temperature by switching off the laser. This laser on-off irradiation cycle was repeated 4 times.

The photothermal conversion efficiency (*η*) of Cys-CuS nanosheets was determined by the following method:^32^ 1 mL of Cys-CuS nanosheet solution (100 mg L^−1^) or pure water was added into a quartz cell. The sample was irradiated with a 1064 nm laser (2 W cm^−2^) for 15 min and cooled for another 15 min with the laser in the off position. Based on the previous report, *η* can be calculated using eqn (1):1
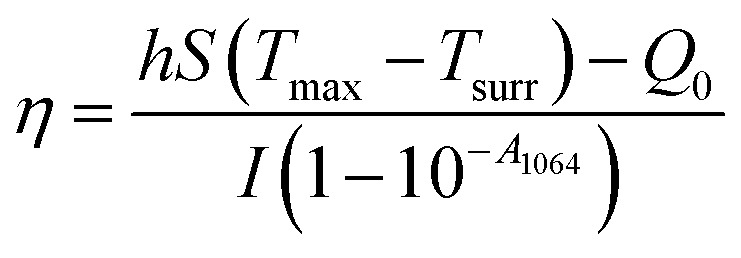
where *T*_max_ is the highest temperature of Cys-CuS nanosheets; *T*_surr_ is the surrounding environment temperature (*T*_max_ − *T*_surr_ = 23.4 °C according to Fig. S1a†); *Q*_0_ is the baseline energy from the sample cell (37.4 mW); *I* represents the power density of the laser (2000 mW cm^−1^); *A*_1064_ (2.192) is the absorbance of Cys-CuS nanosheets at 1064 nm; *S* is the surface area of the sample cell; *h* is heat transfer coefficient, *hS* was obtained using eqn (2):2
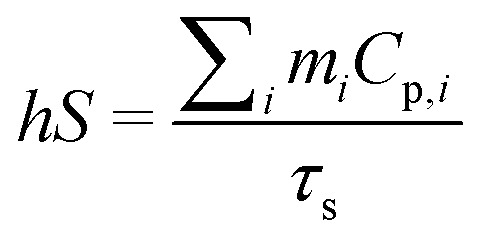


*m*_*i*_ (1 g) is the mass of solvent; *C*_p,*i*_ is the specific heat capacity of solvent (4.2 J g^−1^); *τ*_s_ is the sample system time constant, as followed in eqn (3):

3 *t* = −*τ*_s_ ln *θ*

*θ* is defined as shown in eqn (4); *t*(s) is the cooling period time;4
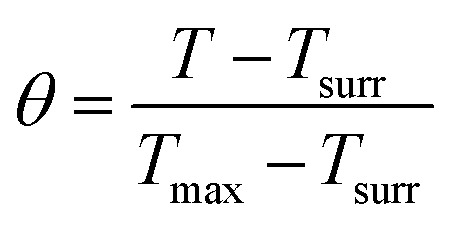


According to eqn (3) and (4), τs is calculated by applying the linear time data from the cooling period *versus* the negative natural logarithm of driving force temperature, as shown in Fig. S1b,† and the result is 270.8 s. Therefore, the heat conversion efficiency (*η*) of Cys-CuS nanosheets at 1064 nm can be calculated as 16.4%.

### Deep-tissue photothermal study

Cys-CuS nanosheet solution (0.8 mg mL^−1^, 1 mL) or pure water (1 mL) were topped with chicken breast muscle of different thicknesses (2, 4, 6, 8, and 10 mm) and then irradiated with 1064 nm laser (1 W cm^−2^) for 2 min. During the irradiation, the temperature of the solution was monitored using an infrared camera.

### Cell cultures

Mouse Leydig (TM3) and Sertoli (TM4) cell lines were cultured in a DMEM culture medium with 2.5% fetal bovine serum and 5% horse serum at 37 °C under 5% CO_2_.

### *In vitro* cytotoxicity

The standard cellular methyl thiazolyl tetrazolium (MTT) assay was performed for the *in vitro* cytotoxicity evaluation of Cys-CuS nanosheets using mouse Leydig (TM3) cells and Sertoli (TM4) cells. Briefly, TM3/TM4 cells were seeded into a 96-well plate with a cell density of 10^4^ per well and cultured for 24 h. Subsequently, the old culture medium was discarded and replaced with a fresh one containing various concentrations of Cys-CuS nanosheet solutions (0, 5, 7.5, 20, 30, and 40 mg L^−1^). After co-culturing for 24 h, the cell viabilities were evaluated by MTT assay and calculated by eqn (5) (*A* is the average absorbance):

5 Cell viability (%) = (*A*_sample_ − *A*_blank_)/(*A*_control_ − *A*_blank_) × 100

### *In vitro* PTT study

For evaluation of the photothermal performance of Cys-CuS nanosheets *in vitro*, TM3/TM4 cells were seeded in 96-well plates (10^4^ per well) and cultured for 24 h. The culture medium was replaced with PBS containing Cys-CuS nanosheets (10 or 20 mg L^−1^) or not. These cells were irradiated with or without a 1064 nm laser (0.8 W cm^−2^) for 5 min and then incubated with fresh DMEM for another 30 min. After that, the cell viability was evaluated *via* MTT assay. Besides, the cells with different treatments (laser 0.8 W cm^−2^ and Cys-CuS nanosheets 20 mg L^−1^) were incubated with Calcein-AM and PI for 15 min and washed with PBS twice. The live/dead cell images were acquired using an inverted fluorescence microscope.

### *In vivo* assessment of toxicity

Animal: all animal studies were conducted with the approval of the Laboratory Animal Ethic Committee of Tianjin Medical University General Hospital (IRB2022-DW-76). Male Kunming mice (8 week-old) were prepared for further applications.

For the evaluation of *in vivo* toxicity of Cys-CuS nanosheets, Kunming mice were intra-testicular injected with or without 100 μL of Cys-CuS nanosheets (1 mg mL^−1^). On the 14th and 60th days, after injection, major organs (heart, liver, spleen, lung, and kidney) were collected and immediately placed into formalin solution for 24 h. Paraffin embedding, routine sections, and staining with hematoxylin and eosin (H&E) were performed based on the standard laboratory histological study procedure.

### NIR-II PTT for male sterilization

Male Kunming mice (8 weeks, 120 mice) were randomly divided into four groups (*n* = 5 for each group at different time points): (i) control; (ii) Cys-CuS; (iii) PBS + NIR-II; (iv) Cys-CuS + NIR-II. The mice were anesthetized by intraperitoneal injection of 10% chloral hydrate. Mice in group (iii) were intra-testicular injected with PBS (100 μL, 10 mM, pH 7.4), and mice in group (ii) and group (iv) were intra-testicular injected with Cys-CuS nanosheets (100 μL, 1 mg mL^−1^). Then, the testes were irradiated with or without 1064 nm laser (1 W cm^−2^) for 40 s. Meanwhile, the temperature changes of testes were recorded with an infrared camera (FLIR E75). After treatments, male mice were sacrificed at different time points (0, 7, 14, and 60 days), and testes and major organs (heart, spleen, liver, lung, and kidney) were harvested for weighting and H&E staining. Besides, blood samples were collected from these groups on the 14th and 60th day after treatment for hormone assay. Levels of testosterone in plasma were measured by ELISA assay.

### Bodyweight measurement and mating tests

After the different treatments mentioned above, the weights of male mice in each group were monitored and recorded every three days. After 14 and 60 days of monitoring, male mice from different groups were caged with two virgin female mice for the mating tests (*n* = 5). The female mouse was separated and fed alone after the successful mating, and the number of pups was recorded.

### Statistical analysis

Student's *t*-test was used for comparison between two groups, and one-way analysis of variance (ANOVA) followed by Tukey's honestly significant difference (HSD) test was applied for comparison among multiple groups (ns means no significant difference, * *p* < 0.05, ** *p* < 0.01, and *** *p* < 0.001). The error bar represents the standard deviation calculated by the square root of variance.

## Results and discussion

### Synthesis and characterizations of Cys-CuS nanosheets

**Fig. 1:**
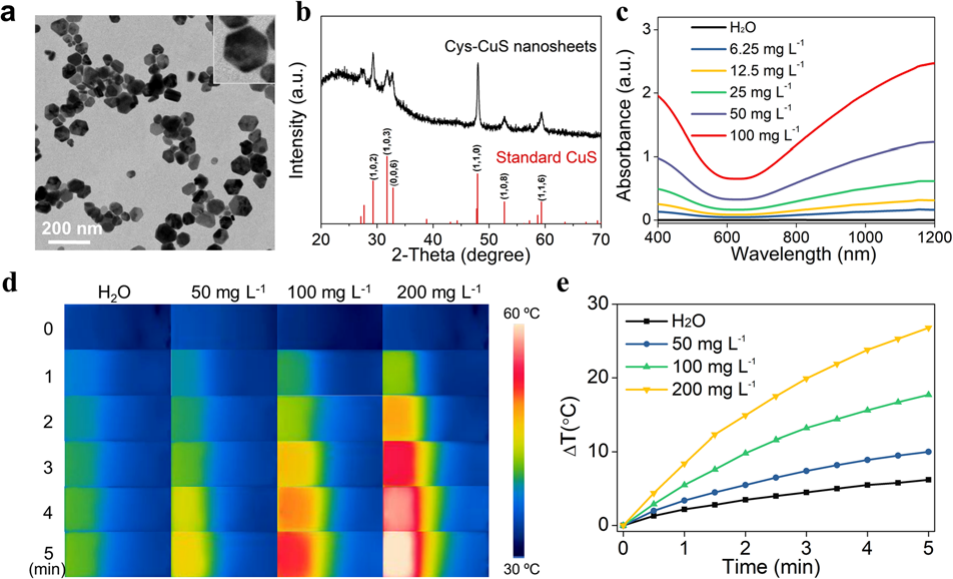
Characterizations and photothermal property of Cys-CuS nanosheets. (a) TEM of Cys-CuS nanosheets (inset picture is a typical polygon shape). (b) XRD spectrum of Cys-CuS and standard CuS (JCPDS card: 06-0464). (c) Absorption spectra of Cys-CuS nanosheet solutions with different concentrations. IR thermal images (d) and temperature curves (e) of pure water and Cys-CuS nanosheet solutions upon irradiation with the 1064 nm laser (2.0 W cm^−2^, 5 min).

Polygon Cys-CuS nanosheets were prepared by a synthetic hydrothermal method. In a typical synthesis, Cys solution was mixed with CuCl_2_ solution to form the Cys–Cu^2+^ complex through the Cu–S bond, followed by adding NaOH aqueous solution and heating at 120 °C to trigger the generation and growth of CuS nanocrystals. As-obtained Cys-CuS displayed a relatively uniform polygon sheet-like structure with a mean diameter of approximately 63 nm (Fig. 1a). The XRD pattern of Cys-CuS nanosheets showed a typical CuS in the hexagonal phase (JCPDS card: 06-0464) (Fig. 1b).^33^ The XPS spectrum shows the chemical states of Cu and S, as shown in Fig. S2a and b,† respectively. In the spectrum of Cu, two distinct peaks at 931.6 eV and 951.5 eV conform to Cu 2p_3/2_ and Cu 2p_1/2_, respectively, and the slight asymmetry in the shape of these two peaks and a small satellite between them revealed the presence of Cu(ii).^33,34^ In the spectrum of S, the binding energies at 162 eV meet with S 2p_3/2_, and 163 eV with S 2p_1/2_ (Fig. S2b†). The hydrodynamic diameter of these nanosheets was measured to be nearly 74 nm (Fig. S3†), and the zeta-potential of aqueous-phase Cys-CuS nanosheets was measured to be −14.1 mV (Fig. S4†).

The stability of Cys-CuS in PBS, normal saline, DMEM, fetal bovine serum, and deionized water was evaluated at different time points. As shown in Fig. S5a,† the mixtures of Cys-CuS in different solutions were relatively stable and homogeneous after standing for 3 days. However, on the 5th and 7th days, there was an obvious precipitate in the mixtures of DMEM, PBS, and normal saline. At the same time, the difference could be observed in the particle size distribution of these mixtures (Fig. S5b†). The particle sizes of Cys-CuS in DMEM, PBS, and normal saline are larger than those in fetal bovine serum and deionized water, which may be due to the relatively high ionic strength in these solvents could cause particle aggregation, thus changing the dispersion behavior of CyS-CuS nanosheets. However, the particle sizes and zeta potential (Fig. S5c†) did not have a time-dependent regular change in any of these solvents during monitoring. According to these results, Cys-CuS nanosheets seem to be less stable in DMEM, PBS, and normal saline for long-time dispersing. It should be noted that a slight precipitate could be observed at the bottom when the Cys-CuS solution was kept standing for some time. However, this precipitate could be quickly redissolved under ultrasonic dispersion (Fig. S6†). Therefore, it is recommended to store the obtained Cys-CuS nanosheets in powder form and prepare them freshly before further application.

As shown in Fig. 1c, Cys-CuS nanosheets displayed a continuous and strong absorption extending from 400 to 1200 nm, particularly in the NIR-II region. Even at an ultralow concentration of 6.25 mg L^−1^, the absorbance of Cys-CuS nanosheets at 1064 nm could reach around 0.143. The extinction coefficient of the proposed Cys-CuS nanosheets at 1064 nm was 21.88 L g^−1^ cm^−1^, which indicated the good absorption ability of Cys-CuS nanosheets in the NIR-II region. Besides, the stability of Cys-CuS nanosheets was further confirmed by the absorption spectra, and there was no obvious difference between the samples stored for various periods (Fig. S7†). These results demonstrated that the Cys-CuS nanosheets possessed good stability under suitable storage conditions and proper usage, which favored further bioapplications.

### Photothermal property of Cys-CuS nanosheets

In view of good absorption in the NIR-II region, further evaluation of the photothermal performance of Cys-CuS nanosheets was performed under irradiation of a 1064 nm laser. Briefly, aqueous solutions of Cys-CuS nanosheets with different concentrations were continuously irradiated with a 1064 nm laser (2 W cm^−2^) for 5 min. As shown in Fig. 1d, e and S8,† the temperature changes of Cys-CuS solutions displayed obvious time-, concentration-, and laser density-dependent. The pure water showed a mild temperature increase by 6 °C, whereas 200 mg L^−1^ Cys-CuS solution exhibited striking temperature elevation by nearly 27 °C under the same irradiation. This indicated that Cys-CuS nanosheets could effectively convert NIR-II light into local hyperthermia, as verified by the good heat conversion efficiency (*η*) of Cys-CuS nanosheets (16.4% at 1064 nm). Besides, considering that photostability played a critical role in maintaining the temperature under light irradiation, the Cys-CuS solution underwent repeated 1064 nm laser irradiation and was measured by a thermocouple probe (Fig. S9†). No significant change was observed in the magnitude of the temperature of the Cys-CuS solution after four cycles of irradiation. The deep-tissue photothermal heating capability was further evaluated in the presence of different thicknesses of chicken breast muscle. Cys-CuS nanosheet solution was added to a cuvette and topped with chicken breast muscle, and the temperature changes were recorded during 1064 nm laser irradiation (Fig. S10†). With the increase in muscle thickness, the maximum temperature changes of the solution gradually decreased. In contrast, the Cys-CuS solution still maintained a better photothermal heating capability than water under the same irradiation conditions. These results confirmed that Cys-CuS nanosheets possessed a relatively high extinction coefficient, good photothermal conversion efficiency, as well as good photothermal stability under a 1064 nm laser, potentiating the high-performance NIR-II light-responsive PTT.

### *In vitro* NIR-II PTT assessment

**Fig. 2:**
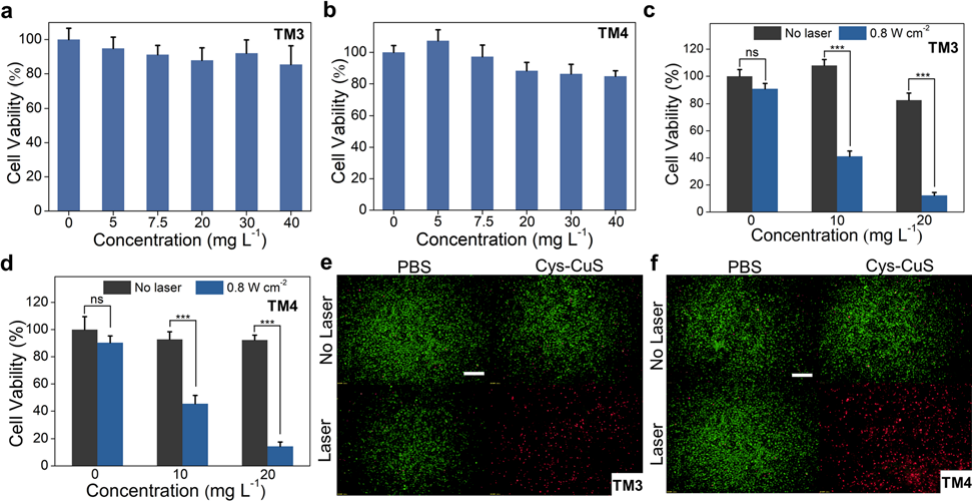
Cytotoxicity and *in vitro* NIR-II PTT of Cys-CuS nanosheets. Cell viability of TM3 (a) or TM4 cells (b) after incubation with or without various concentrations of Cys-CuS nanosheets for 24 h. Relative viabilities of TM3 (c) or TM4 cells (d) treated with or without Cys-CuS nanosheets and 1064 nm laser (0.8 W cm^−2^) for 5 min (ns: no significant difference, *** *p* < 0.001). Fluorescence images of TM3 (e) or TM4 cells (f) with live/dead staining after different treatments (laser 0.8 W cm^−2^ and Cys-CuS nanosheets 20 mg L^−1^). The scale bar is 500 μm.

An ideal PTA should satisfy the toxicity requirement in biological applications. Thus, the cytotoxicity of Cys-CuS nanosheets was evaluated before photothermal therapy by a standard MTT assay (Fig. 2a and b). Negligible cytotoxicity was observed in both mice Leydig (TM3) cells and Sertoli (TM4) cells after incubation with different concentrations of Cys-CuS nanosheets. The relative viabilities of both TM3 and TM4 cells were retained up to 85% with a concentration of Cys-CuS at 40 mg L^−1^. To further verify the NIR-II PTT effect of Cys-CuS nanosheets *in vitro*, we evaluated its phototherapy efficiency against TM3 cells or TM4 cells *via* MTT assay and live/dead staining. The TM3 or TM4 cells were previously treated with or without different concentrations of Cys-CuS nanosheets or/and 1064 nm laser irradiation. As shown in Fig. 2c and d, laser irradiation or Cys-CuS nanosheets alone displayed a negligible influence on cell viability. In comparison, the cell viability decreased evidently (lower than 20%) when TM3 or TM4 cells were treated with both Cys-CuS nanosheets (20 mg L^−1^) co-incubation and 1064 nm laser irradiation (0.8 W cm^−2^) for only 5 min. Besides, the typical groups were prepared for live/dead cell staining to intuitively observe the cell viabilities after PTT through the co-staining by Calcein-AM (green for live cells) and PI (red for dead cells) as shown in Fig. 2e and f, respectively. Compared with the control group, TM3 or TM4 cells treated with only Cys-CuS nanosheets or 1064 laser irritation displayed strong green emissions, reflecting that most cells were alive. While nearly all cells were dead in the group treated with Cys-CuS nanosheets and laser irradiation with a full view of red emission. These results demonstrated that Cys-CuS nanosheets could efficiently induce death in Sertoli cells and Leydig cells *via* photothermal ablation under 1064 nm laser irradiation. In mouse testis, Sertoli cells are crucial in the differentiation of germ cells in spermatogenesis, and Leydig cells can produce testosterone to influence reproductive and sexual functions. The destruction of these cells driven by Cys-CuS nanosheets-mediated NIR-II PTT is a disaster for mouse reproduction.

### *In vivo* NIR-II PPT for male sterilization

**Fig. 3:**
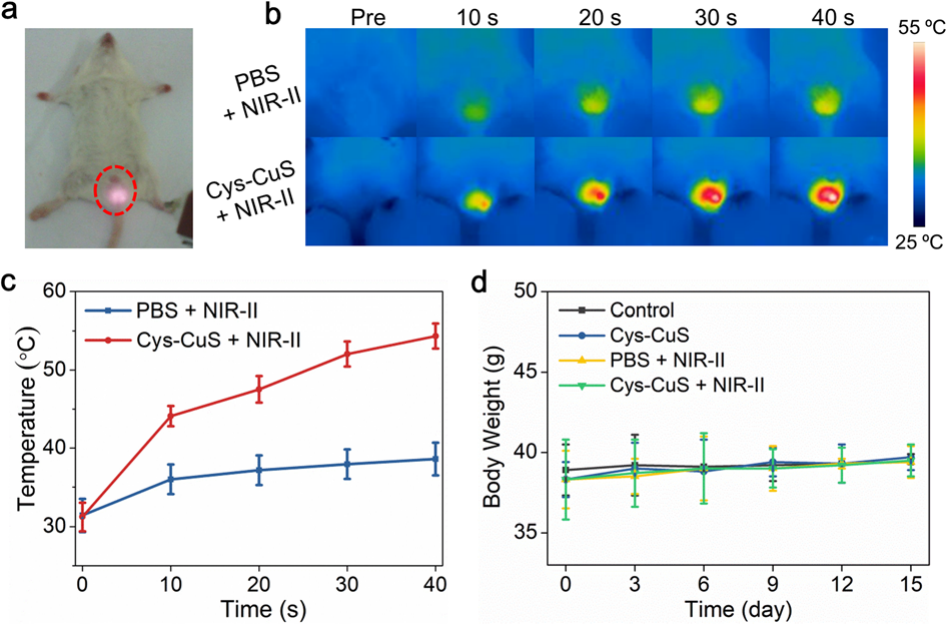
Cys-CuS nanosheets-mediated NIR-II PTT for male sterilization *in vivo*. Male Kunming mice were randomly divided into four groups: (i) control; (ii) Cys-CuS (100 μL, 1 mg mL^−1^); (iii) PBS (100 μL) + NIR-II (1064 nm, 1 W cm^−2^); (iv) Cys-CuS + NIR-II (100 μL, 1 mg mL^−1^ + 1064 nm, 1 W cm^−2^). Typical photographs (a), thermal infrared images (b), and temperature changes (c) of testes in group (iii) and group (iv) at different time intervals under the irradiation of 1064 nm laser (1 W cm^−2^). (d) The body weight change profiles of mice in different groups after treatments.

Encouraged by *in vitro* results, we next evaluated the *in vivo* photothermal performance of the CyS-CuS nanosheets as a light-driven sterilant. Male Kunming mice were divided into four groups randomly for different treatments: (i) control; (ii) Cys-CuS; (iii) PBS + NIR-II; (iv) Cys-CuS + NIR-II. After intra-testicular injection of 100 μL Cys-CuS nanosheet solution (1 mg mL^−1^) or PBS, the testis regions underwent 1064 nm laser irradiation (1 W cm^−2^, 40 s) (Fig. 3a). Temperature changes in the testis and thermographic images were recorded every 10 s during the irradiation (Fig. 3b and c). The average temperatures of the testes slightly increased by 7 °C, and remained around 38 °C in mice of the PBS + NIR-II group. In contrast, the average temperature of testes treated with Cys-CuS nanosheets elevated rapidly to nearly 44 °C after 10 s irradiation and continuously increased to nearly 54 °C at the end of irradiation. This heating process was closely associated with the satisfactory photothermal conversion efficiency and extinction coefficient of Cys-CuS nanosheets. After treatments, the mice were housed and monitored for future experiments. During the experiment and daily monitoring period, the mice in these groups did not show any abnormal behaviors or death. As shown in Fig. 3d, the body weights of the mice in these groups did not exhibit significant differences.

**Fig. 4:**
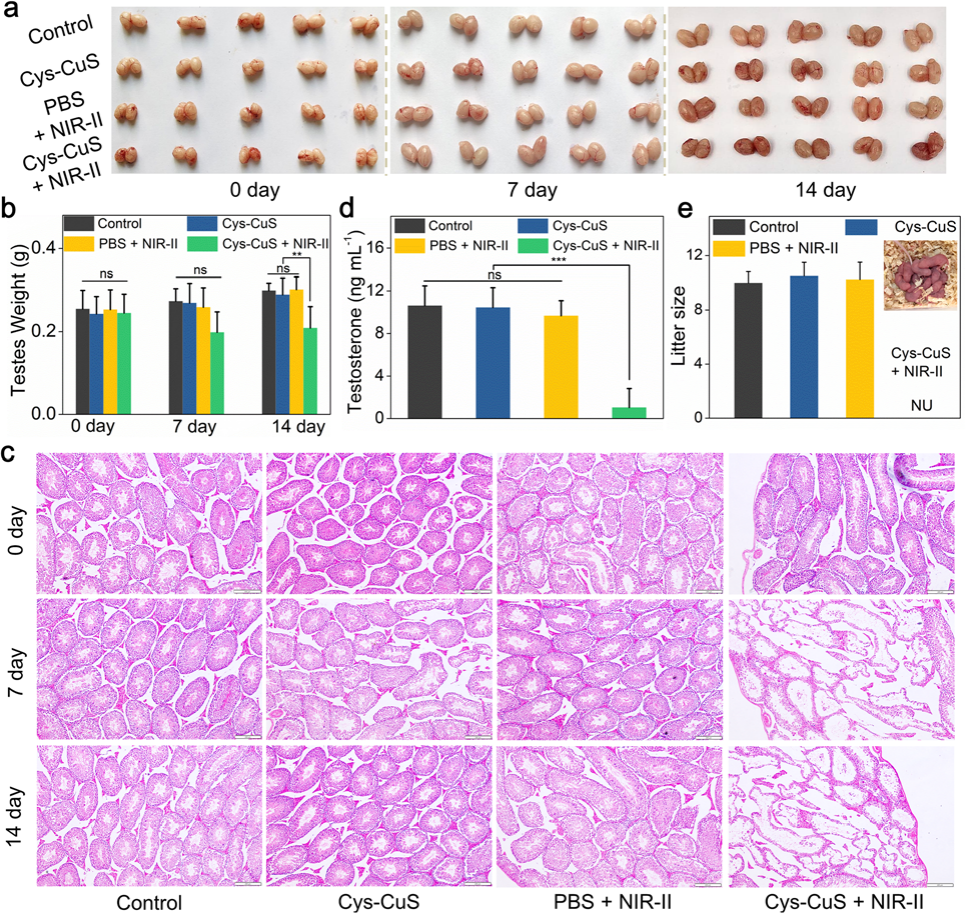
Cys-CuS nanosheets-mediated NIR-II PTT for male sterilization *in vivo*. Photographs of testes (a) and testis weights (b) in different groups on the 7th day and 14th days of the post-treatment. (c) H&E staining of testes in different groups on the 7th day and 14th days of the post-treatment (scale bar: 200 μm). (d) Plasma levels of testosterone in mice on the 14th day of the post-treatment. (e) The average number of pups/pregnant females after mating with male mice in different groups (NU represents no pups was observed). Ns: no significant difference, ** *p* < 0.01, *** *p* < 0.001.

As reported in previous studies, hyperthermia could induce morphological changes in testes and seminiferous tubules.^7,10,11^ Contraceptive efficacy of Cys-CuS nanosheets was evaluated in terms of testicular function and fertility evaluation. Experimental mice in different groups were sacrificed on the 7th, 14th, and 60th days of the post-treatment, and then testes were harvested, weighed, sectioned, stained, and analyzed. As shown in Fig. 4a, no visible difference could be observed in the size of testes among these groups at any time point. Actually, the testes in Cys-CuS + NIR-II group showed a slight weight loss (Fig. 4b) on the 14th and 60th day of post-treatment, which may be due to the partly destroyed testes by hyperthermia. Histological analysis (Fig. 4c) was then carried out to evaluate the structural changes of the testes. It was clearly revealed that seminiferous tubules of testes in group (iv) were partially destroyed compared to the other three groups. These results confirmed the successful destruction of the morphology of testes with NIR-II PTT, and the treatment by laser or Cys-CuS injection only will not destroy the structure of the testes. In addition, we further assessed the testis function in these groups by testosterone and fertility assessments. As we all know, testosterone is a hormone primarily produced by Leydig cells in the testis, and its hormone level in plasma could reflect the damage degree of the testes. As shown in Fig. 4d and S11,† the plasma level of testosterone in group (iv) on the 14th day of the post-treatment was nearly 10-fold lower than those in the other three groups with statistical differences. At the 60th day post-treatment, the mean testosterone levels in group (iv) showed an obvious downward trend, but there are no statistical differences between these four groups. Besides, fertility assessments were carried out on the 14th and 60th days after treatment. Each male mouse from different groups was caged with two virgin female mice. As shown in Fig. 4e, the pregnant female mice in the control group, Cys-CuS, and PBS + NIR-II gave birth to almost the same number of pups. No obvious morphological defects or abnormal behaviors could be observed in these pups. While the female mice in the Cys-CuS + NIR-II group had no pups at all, which reflected that male mice in this group had totally or partly lost their reproductive capacity. These data indicated that both morphology and functions of the testis were destroyed by treatment with Cys-CuS plus 1064 nm laser irradiation. Besides, the major organs (heart, liver, spleen, lung, and kidney) of Kunming mice were harvested and stained after intra-testicular injection with PBS or Cys-CuS nanosheet solution. The H&E staining images (Fig. S12†) displayed that no significant histological abnormalities were observed. Taken together, Cys-CuS nanosheets are a promising NIR-II light-responsive nanoknife for safe permanent male sterilization, which could greatly improve the efficiency of sterilization surgery.

## Conclusions

In summary, to achieve a simple, controllable, effective, and minimally invasive permanent male sterilization method, we developed a biocompatible NIR-II light-responsive “nanoknife” Cys-CuS nanosheet through a facile one-pot hydrothermal synthesis. During the synthetic process, biocompatible Cys was fully utilized as both template and sulfur provider. The obtained polygon-like nanosheets showed strong and broad absorption in the NIR-II region, good extinction coefficient, and photothermal conversion efficiency, and satisfied deep-tissue light response capacity under 1064 nm laser irradiation. Given these merits, Cys-CuS nanosheets successfully enabled the biosafe NIR-II PTT for permanent male sterilization *in vivo* for the first time. We believe that these biomimetic synthetic Cys-CuS nanosheets are promising NIR-II light-responsive nanoknife for permanent male sterilization in animals.

## Author contributions

The manuscript was written through the contributions of all authors. All authors have given approval to the final version of the manuscript.

## Conflicts of interest

There are no conflicts to declare.

## Supplementary Material

NA-005-D3NA00189J-s001
