# Supplementary material for: A biocompatible NIR-II light-responsive nanoknife for permanent male sterilization
Source: Nanoscale Adv. 2023 Aug 16;5(18):5029–35. doi: 10.1039/d3na00189j (PMC10496908; doi:10.1039/d3na00189j)
Supplement: NA-005-D3NA00189J-s001 [file NA-005-D3NA00189J-s001.pdf]

Electronic Supplementary Material (ESI) for Nanoscale Advances.
This journal is © The Royal Society of Chemistry 2023

A biocompatible NIR-II light-responsive nanoknife for permanent male sterilization

Haoyu Wang,[a,b,‡] *Xiaomeng Yue,*[c,‡] *Huanhuan Wu,* [a] *Yeda Wan,*[c] *Yujie Tong,*[a] *Yang Zhao,*[d] *Yijun Li,*[e] *and Jinbin Pan*[a,*]

a. Department of Radiology, Tianjin Key Laboratory of Functional Imaging, Tianjin Medical University General Hospital, Tianjin 300052, China.

b. Department of Radiology, Affiliated Hospital of Inner Mongolia Medical University, Hohhot 010050, China.

c. Ultrasonic Diagnosis and Treatment Department, National Clinical Research Center of Cancer, Tianjin Cancer Hospital Ariport Hospital, Tianjin 300052, China.

d. Department of Radiology, The Second Hospital of Tianjin Medical University, Tianjin 300211, China.

e. Inner Mongolia Medical University, Hohhot 010050, China.

‡ These authors contributed equally to this article.

*Corresponding authors. E-mail:panjinbin@tmu.edu.cn

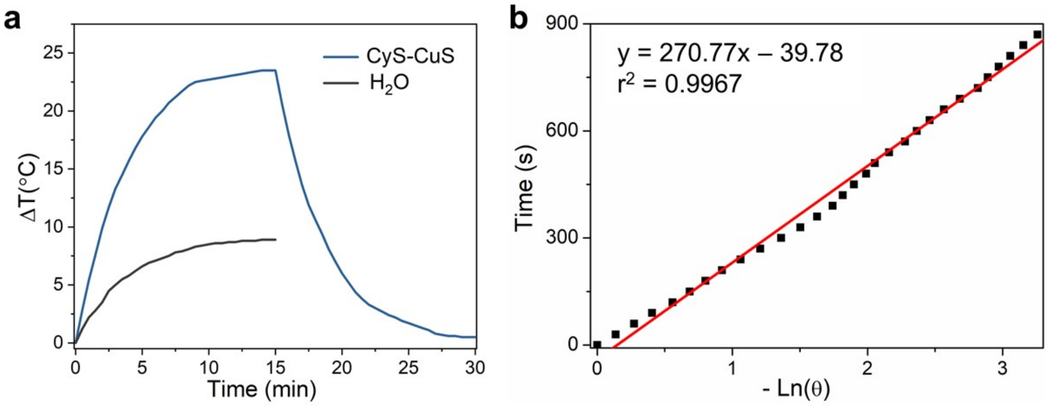

Figure S1. Temperature evolution curves of Cys-CuS nanosheets in a 1064 nm laser on/off cycle. a) Temperature profile of Cys-CuS solution (100 mg L^{-1}) under the irradiation of a 1064 nm laser (2.0 W cm^{-2}) for 15 min, and then the laser was turned off. b) Plot of the cooling time versus -Ln(θ) obtained from the cooling stage in (a).

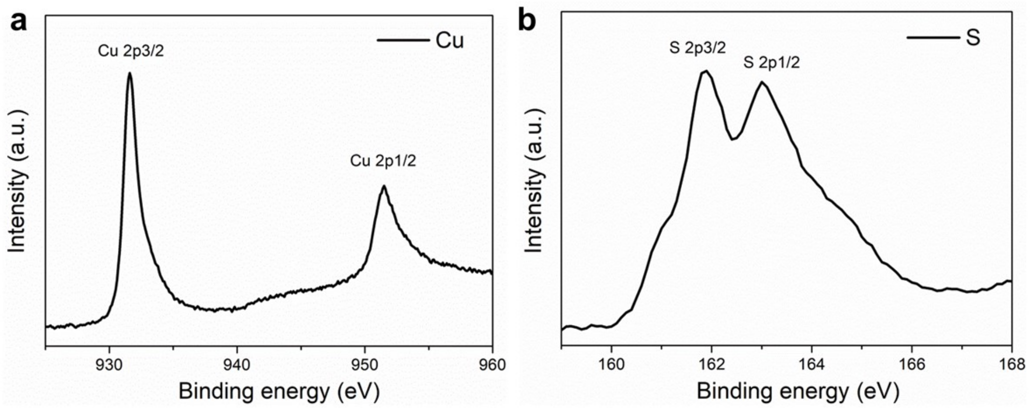

Figure S2. XPS spectra of (a) Cu 2p and (b) S 2p in Cys-CuS nanosheets.

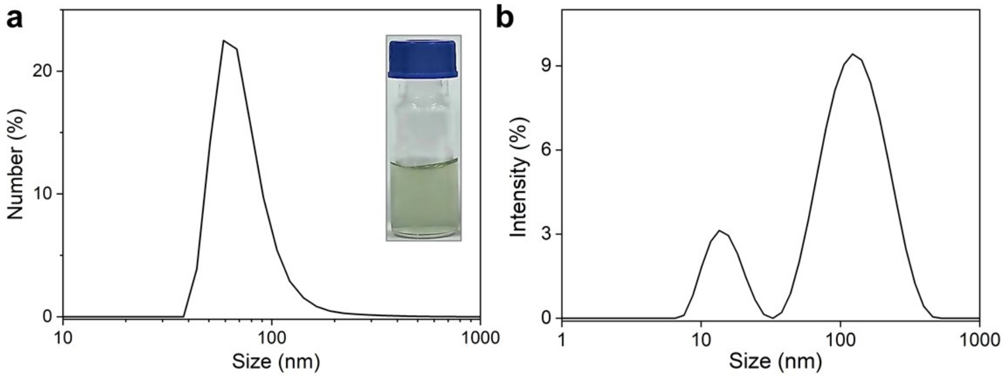

Figure S3. Hydrodynamic size of Cys-CuS nanosheets measured by DLS. The insert photograph is Cys-CuS aqueous solution (0.04 mg mL^{-1}).

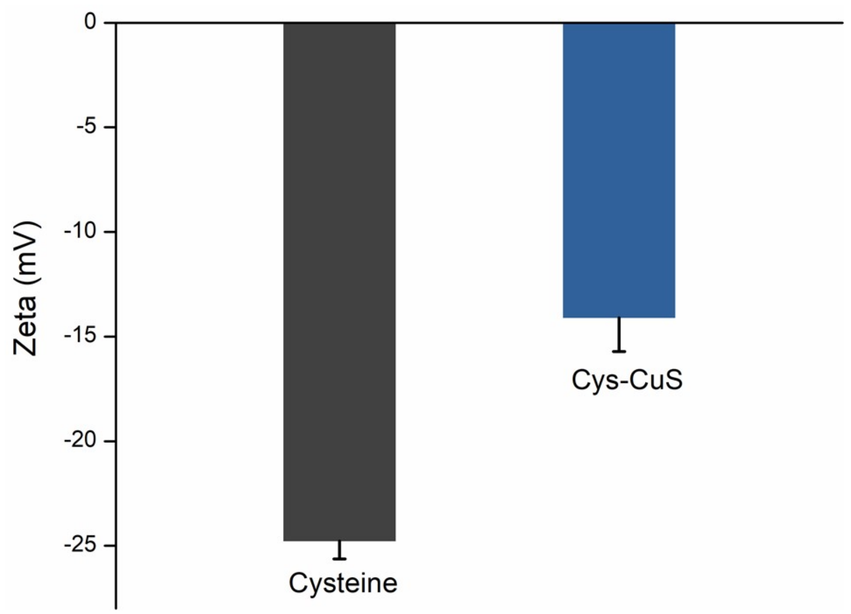

Figure S4. The zeta potential of Cystine and Cys-CuS nanosheets.

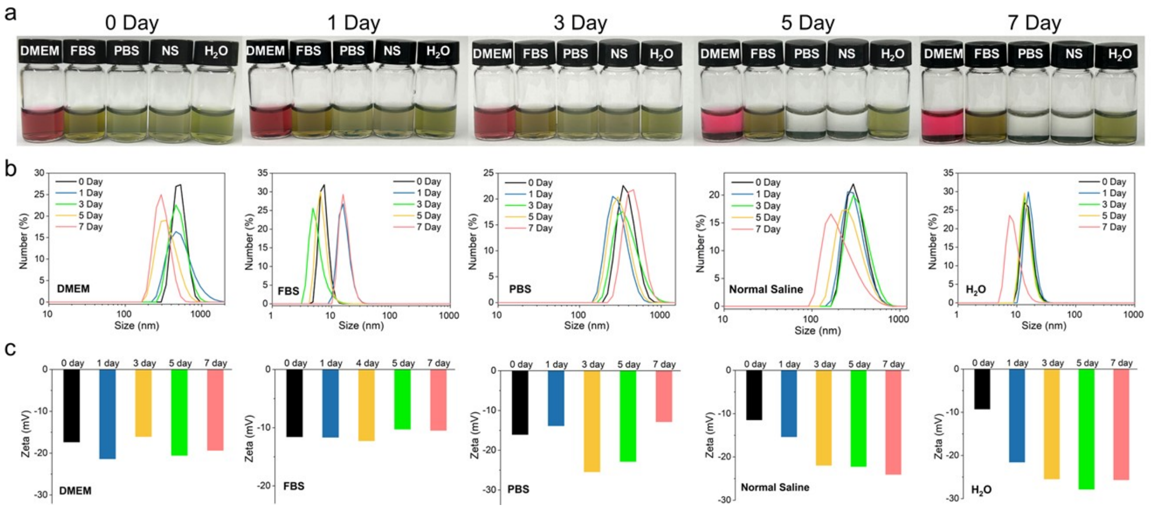

Figure S5. The photograph (a), DLS (b) and zeta potential (c) of Cys-CuS in PBS, normal saline, DMEM, fetal bovine serum (FBS), and deionized water at different time points (0, 1, 3, 5, 7 days)

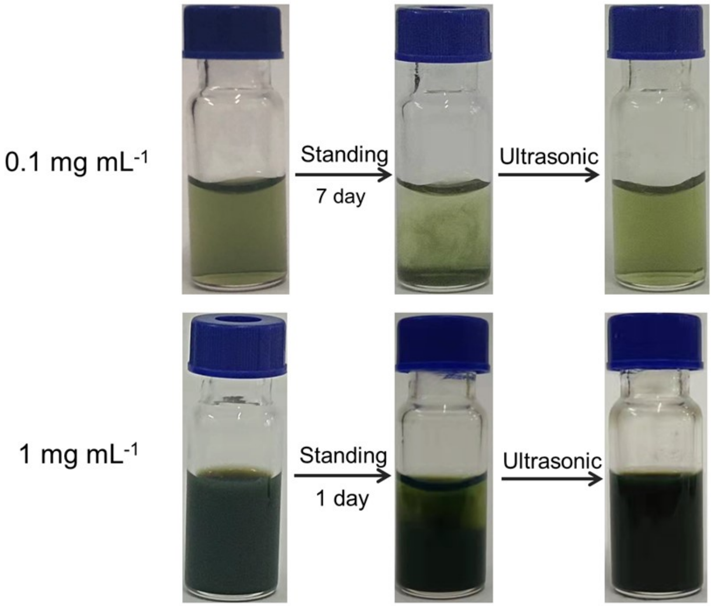

Figure S6. Photographs of Cys-CuS nanosheets solutions after standing for some time.

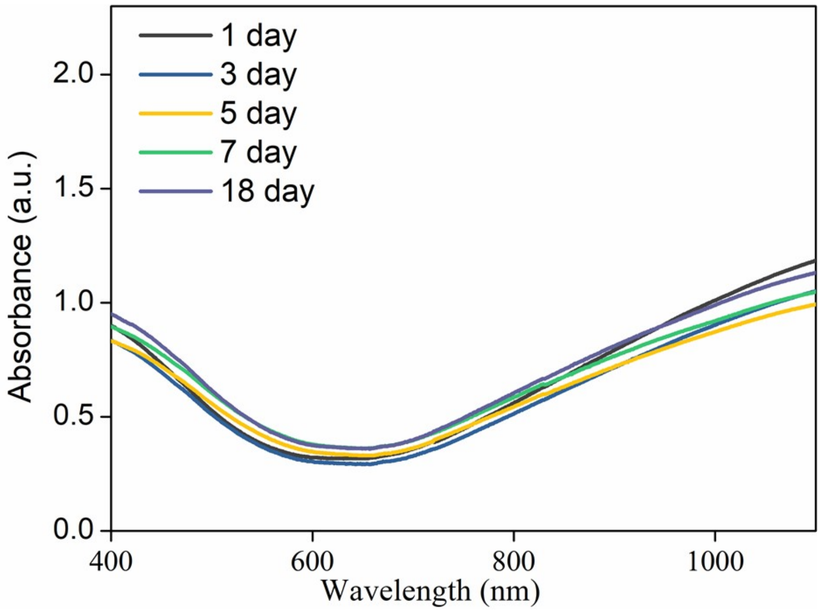

Figure S7. The UV-vis-NIR absorption spectra of Cys-CuS nanosheets (50 mg L^{-1}) solutions after store for different durations.

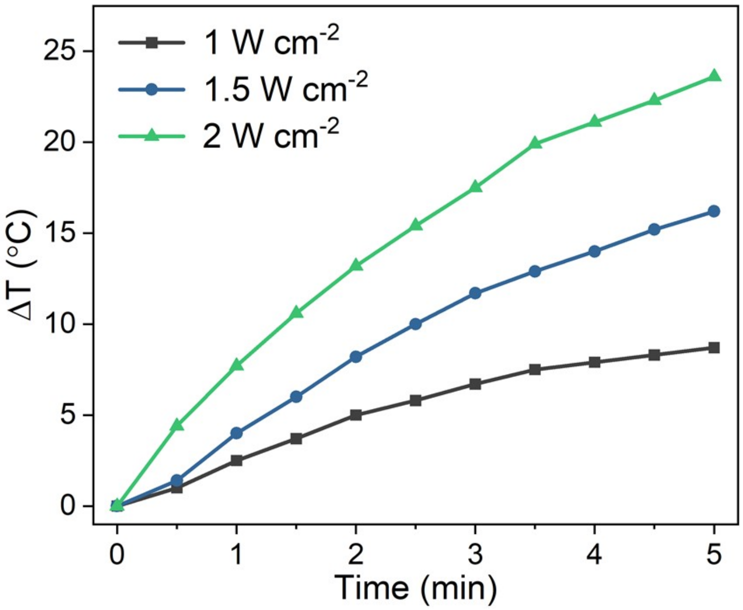

Figure S8. Temperature curves Cys-CuS nanosheets (200 mg L^{-1}) solutions upon irradiation of 1064 nm laser (1, 1.5, 2.0 W cm^{-2}, 5 min).

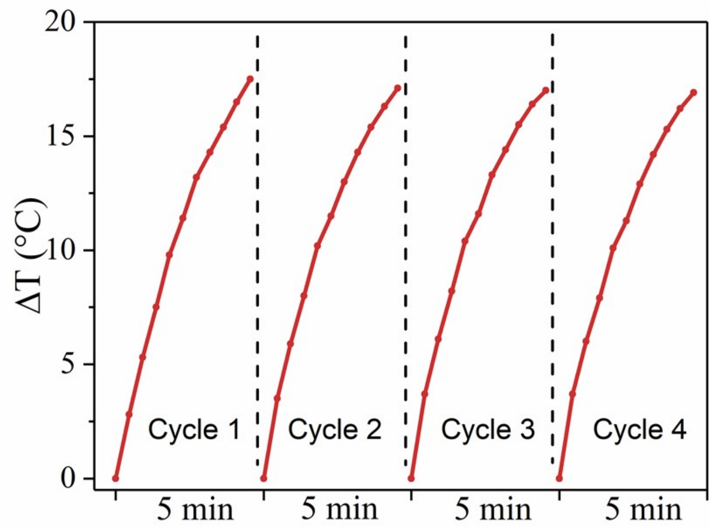

Figure S9. Photothermal stability of Cys-CuS nanosheets (100 mg L^{-1}) under cyclic irradiation of 1064 nm laser (2 W cm^{-2}).

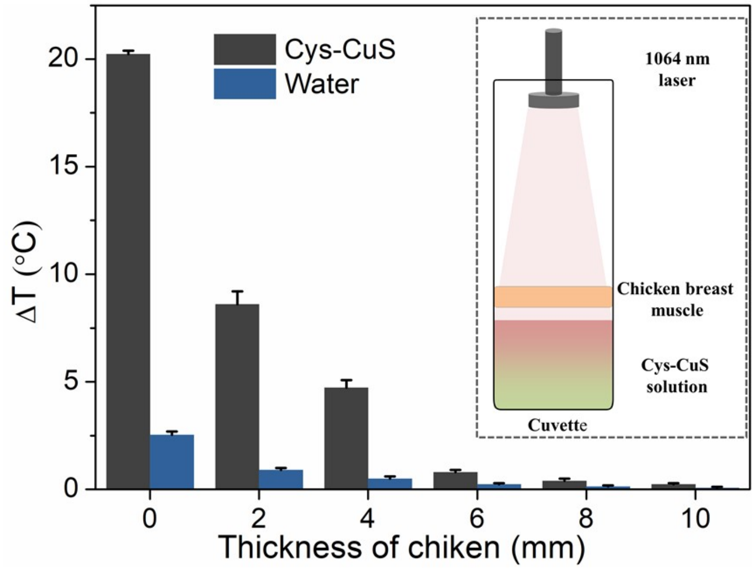

Figure S10. Deep-tissue photothermal study of Cys-CuS nanosheets (80 mg L^{-1}, 1 mL) covered by different thicknesses of chicken breast muscle (2, 4, 6, 8, 10 mm) under a 1064 nm laser irradiation (1 W cm^{-2}, 2 min).

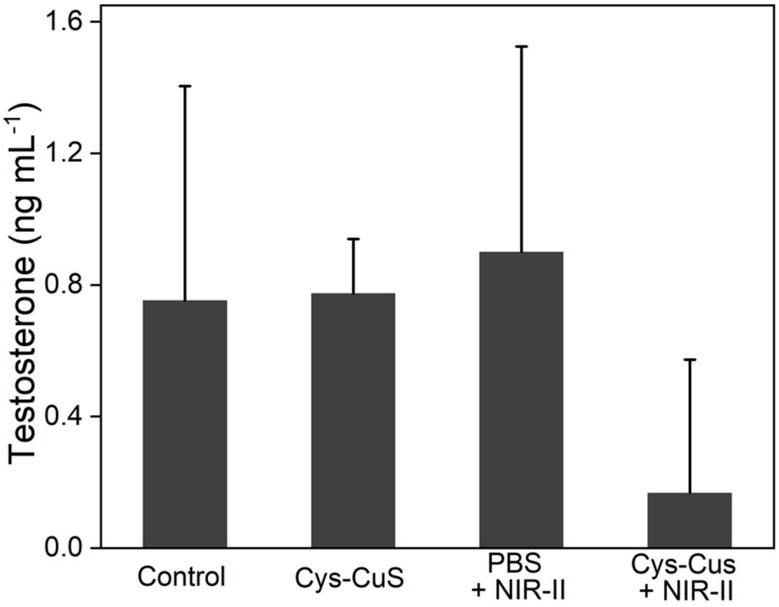

Figure S11. Plasma levels of testosterone in mice at 60th day post treatments.

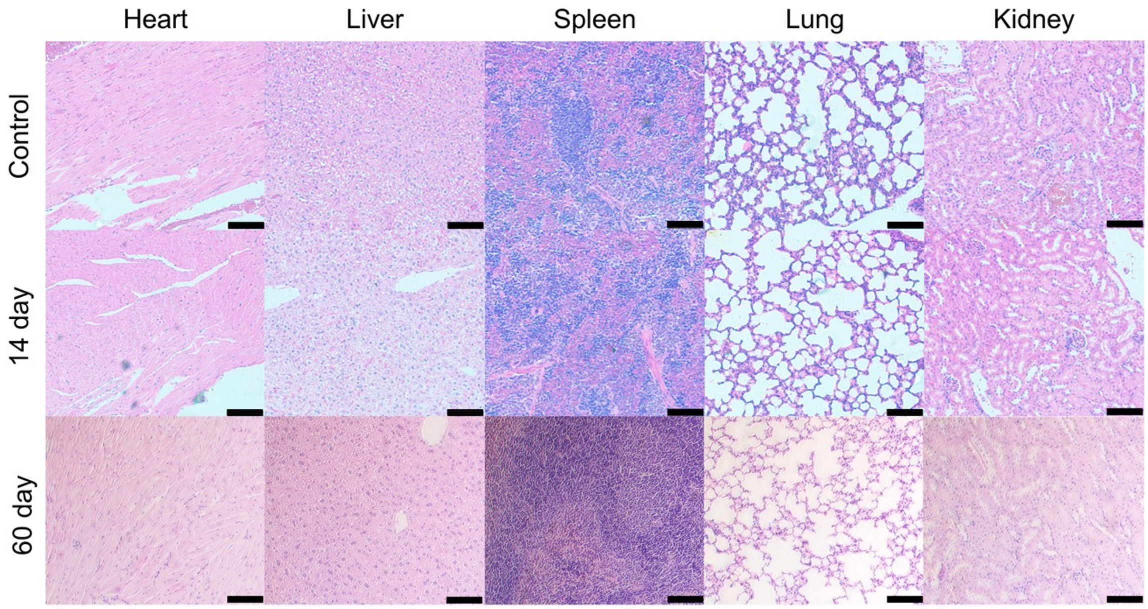

Figure S12. H&E staining of major organs from mice after intra-testicular injection with or without Cys-CuS nanosheets (100 μL, 100 mg L^{-1}). (Scale bar: 100 μm)
